# Development of Crystalline Cu_2_S Nanowires via a Direct Synthesis Process and Its Potential Applications

**DOI:** 10.3390/nano10020399

**Published:** 2020-02-24

**Authors:** Chih-Yen Chen, Jian-Ru Jiang, Wen-Shuo Chuang, Ming-Song Liu, Sheng-Wei Lee

**Affiliations:** 1 Department of Materials and Optoelectronic Science, National Sun Yat-Sen University, Kaohsiung 804, Taiwan; 2 Institute of Materials Science and Engineering, National Central University, Taoyuan 320, Taiwan

**Keywords:** Cu_2_S nanowires, nanomaterials, vacuum, field emission, photocatalytic degradation, environmentally friendly approach

## Abstract

Large-scale and uniform copper(I) sulfide (Cu_2_S) nanowires have been successfully synthesized via a cheap, fast, easily handled, and environmentally friendly approach. In addition to the reductive properties of the biomolecule-assisted method, they also have a strong shape- or size-directing functionality in the reaction process. The field-emission properties of the Cu_2_S nanowires in a vacuum were studied by the Folwer–Nordheim (F–N) theory. The Cu_2_S nanowires have a low turn-on field at 1.19 V/μm and a high enhancement factor (β) of 19,381. The photocatalytic degradation of Cu_2_S nanowires was investigated by the change in the concentrations of rhodamine B (RhB) under UV illumination. These outstanding results of Cu_2_S nanowires indicate that they will be developed as good candidates as electron field emitters and chemical photocatalysts in future nanoelectronic devices.

## 1. Introduction

Over the past decades, a variety of nanostructured semiconductors with well-controllable size, shape, and composition have been widely developed and applied in many kinds of fields, such as catalysis, size-effect, photocatalytic, and biomaterial applications [1,2,3]. Some important studies have indicated that the properties of nanomaterials depend on their morphologies and sizes, so that their chemical and physical properties are quite different from each other in bulk materials [4,5,6,7]. Among various metal chalcogenide semiconductor nanostructures, the copper(I) sulfide (Cu_2_S) nanostructure is regarded as a potential material for future applications in optoelectronic devices [8,9,10]. Cu_2_S is an excellent p-type semiconductor material with an indirect bandgap of 1.2 eV, and it could be applied in photovoltaics [9], nanoscale sensors [11,12], and cathodes for lithium-ion batteries [13,14,15]. 

A lot of efforts have been devoted in recent years to developing semiconductor photocatalysts with high photocatalytic activities for environmental protection [8,11,13,14,15]. Most photocatalytic degradation studies have focused on the use of nano-crystalline titanium oxides (TiO_2_) [16,17], but their photocatalytic efficiency is quite poor, especially under visible light irradiation. In this respect, metal chalcogenide semiconductors have suitable photocatalytic activities under solar irradiation, so they might be good alternatives for replacing the TiO_2_. A variety of different Cu_2_S nanostructures such as nanowires, nanoplates, and nanocrystals have been synthesized using diverse routes [11,12,13,14,15]. Previous studies have developed several synthesis routes for the growth of Cu_2_S nanowire arrays, such as thermal evaporation processes and autoclave-assisted hydrothermal processes [18,19,20]. However, we have reviewed these synthesis methods carefully, but many of these are achieved with toxic chemicals. Many of these are strictly regulated because they are toxic, bio-accumulative, disruptive to hormones, and carcinogenic.

In this study, large-scale and uniform free-standing semiconducting copper(I) sulfide (Cu_2_S) nanowire arrays have been successfully synthesized on copper substrates via a fast, easily handled, and environmentally friendly approach. The innovative reduction method, which combines ethylenediamine (En) and hydrazine in an alkali solution, is demonstrated to be effective in synthesizing copper nanowires. Furthermore, Cu_2_S nanowires were investigated experimentally in detail for their filed emitted electronic and photocatalytic properties. A photocatalyst of Cu_2_S structures was investigated by photocatalytic degradation of rhodamine B (RhB) under UV illumination. The previous results indicate that surface area would play a significant factor in the efficiency of photocatalysis, since photocatalytic reaction occurred on the surface [9,11,12]. On the other hand, the Cu_2_S nanowires also show a low turn-on field (1.19 V/μm) and high field enhancement factor (β = 19,381). A smaller cross-sectional area of an emitter will enhance its field-emission performance. Because the tip of the free-standing nanowire has a smaller cross-sectional area, our Cu_2_S nanowires hence have better filed emission properties than other nanoscale-emitting materials or the same bulk crystalline solids in the previous reports [18,21,22]. These availabilities of Cu_2_S nanowires will not only enable new types of applications, but also allow the performance of photoelectricities to be enhanced in future devices.

## 2. Materials and Methods

### 2.1. Synthesis of Cu_2_S Nanowire Structure

In a typical procedure, 10 g sodium hydroxide (NaOH, ≥98%, Sigma-Aldrich, St. Louis, MO, USA) were dissolved in 40 mL distilled water and heated to 70 °C. At a constant 70 °C, 1 g L-Cystine, 7 mL ethylenediamine (≥99%, Sigma-Aldrich), and 40 μL hydrazine (98%, Sigma-Aldrich) were added into the solution. The cleaned copper foil (Nan-ya Plastics Corporation) was then placed into the mixture for 10 h, and the final product was a black film on the Cu substrate. The products on the copper substrate were washed several times with ethanol and Deionized water (DI water), and then dried in an air atmosphere.

### 2.2. Microstructure Characterization

Several precision facilities were thoroughly used for the morphological characterization of the Cu_2_S nanowires. The microstructure and chemical composition were characterized by using a field-emission scanning electron microscope (FE-SEM; Quanta 200, FEI Company, Hillsboro, OR, USA) equipped with an energy-dispersive X-ray spectrometer. High-resolution lattice images were obtained with an ultrahigh vacuum high-resolution transmission electron microscope (HRTEM, JEM-2000FX II, JEOL, Ltd., Tokyo, Japan), operating at 200 keV with a point-to-point resolution of 0.14 nm. X-ray diffraction (XRD, D2 Phaser, Bruker Corporation, Karlsruhe, Germany) was used to characterize the crystal structure of the Cu_2_S nanowires over copper foil, recorded in a Bruker D8A diffractometer by using Cu Kα radiation. It was operated at 40 kV and 40 mA with a scan rate of 0.04° per step.

### 2.3. Measurement of Photodegradation of RhB

The photocatalytic performance of Cu_2_S was evaluated by the degradation of Rhodamine B (RhB, HPLC, Sigma-Aldrich) under UV irradiation using a 200 W Mercury–Xenon (HgXe) arc lamp bulb. Degradation tests were performed by using films with geometrical surfaces of 0.9 × 0.9 cm^2^, immersed in 3 mL RhB solution with the initial concentration of 5 μM. At various irradiation times, the UV-visible absorption spectrum of the solution was measured by using a spectrophotometer (Evolution 60S, Thermo Scientific, Waltham, MA, USA), and the concentration was estimated by the integration of the absorption peak.

### 2.4. The Electron Field Emission Measurements

The field-emission output of Cu_2_S nanowires was carried out in a vacuum chamber under the pressure of 1 × 10^–7^ torr at room temperature. The measurement distances between the anode and the emitting surface of the Cu_2_S nanowires were controlled in 100 μm. The GDM-8246 (GW-Instek Ltd.) instrument was used to measure the current density (I) and electric field (E) characteristics. It was necessary to run over emission cycles for 50 times in order to obtain stable and reproducible I–E characteristics.

## 3. Results 

### 3.1. Growth and Structural Characterizations of Cu_2_S Nanowires

By utilizing the combination of cystine, a compound which has the formula (SCH_2_CH(NH_2_)CO_2_H)_2_, and metal chelating chemistry, successful synthesis of Cu_2_S nanowires on the copper substrate was based on a biomolecule-assisted method. Cystine is an ordinary dimeric amino acid formed by the oxidation of two cysteine molecules that covalently link to make a disulfide bond. The disulfide link is readily reduced to give the corresponding thiol cysteine when it is heated. As a sulfur-containing amino acid, L-cysteine has been also proposed as a structure-directing agent in the synthesis of metal sulfide nanostructures, such as CdS and Bi_2_S_3_ [23,24,25]. In this study, L-cystine was utilized not only as the structure-directing molecule, but also as a sulfur source to prepare the metal sulfide nanostructures. This process is so convenient that Cu_2_S nanowires do not need to be synthesized in a common high-pressure autoclave. 

**Figure 1:**
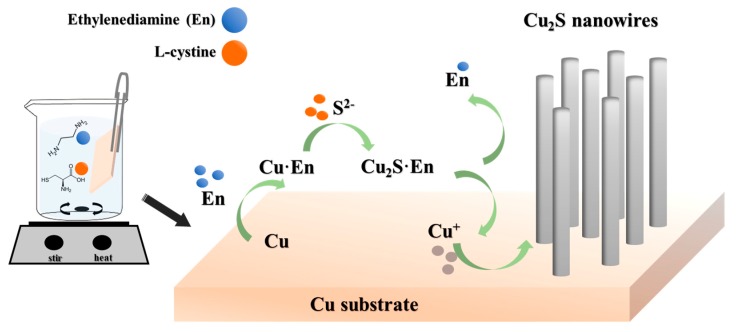
The growth mechanism of the copper(I) sulfide (Cu_2_S) nanowire arrays.

Figure 1 illustrates a possible chemical reaction pathway and growth mechanism of the Cu_2_S nanowire arrays in experiments. The ethylenediamine molecules have a strong coordinating ability and function in metal chelation or in mediating the electron transfer in the reaction system. When the clean copper substrate was placed into the mixture, the ethylenediamine could play a role in activating the surface of the metal substrate and in electron transfer in the reacting system [26,27]. On the other hand, the stability of the Cu-ethylenediamine (Cu–En) metal complex was decreased by increasing the temperature, and a sulfide ion (S^2−^) was produced from the dissociation of cysteine so that S^2−^ could react quickly with the Cu–En complex. The ethylenediamine molecules of the unstable inorganic–organic complex of Cu_2_S–En molecular precursors eventually disappeared [28,29,30], resulting in the formation of Cu_2_S nanocrystals, which are located on the copper substrate in Figure 1.

**Figure 2:**
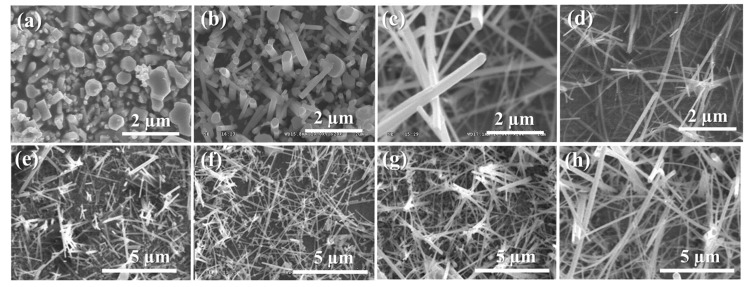
SEM images of as-prepared free-standing Cu_2_S nanowires grown under different reaction conditions. For the first four conditions, (**a**) 5, (**b**) 7.5, (**c**) 10, and (**d**) 20 g NaOH are added with 1 g cysteine and 7 mL ethylenediamine at 70 °C for 10 h. The other four conditions 10 g NaOH, 1 g cysteine, and 7 mL ethylenediamine at 70 °C are controlled for (**e**) 1, (**f**) 3, (**g**) 5, and (**h**) 10 h.

In order to understand the effects of the different reaction conditions on the growth of the free-standing Cu_2_S nanowires, a series of experiments have been carried out by examining the aging time, the amount of sodium hydroxide, and the volume of ethylenediamine. Figure 2 shows a series of low-magnification SEM images of the as-synthesized product grown on the copper substrate. It is well known that synthesizing a Cu–En metal complex results from a copper ion and a ligand of ethylenediamine solution because of a Lewis acid–base interaction. The sulfidation rate has to be studied experimentally in order to obtain high regularity for the Cu_2_S nanowires. The high pH value in the alkali condition is also essential to let the sulfur anions diffuse to the copper substrate slowly and to prevent the rapid sulfidation rate of the copper substrate. Furthermore, a chemical agent of sodium hydroxide is added, which can maintain a strong alkaline environment with a high pH value and also accelerates the dissolution of cystine in the solution. Figure 2a–d shows the morphologies of Cu_2_S nanowires grown in various amounts of alkali solution by changing the amounts of sodium hydroxide (NaOH) from 5, 7.5, and 10 g to 20 g with 1 g cysteine and 7 mL ethylenediamine at 70 °C for 10 h. The other four SEM images of as-prepared Cu_2_S nanowires grown with 10 g NaOH, 1 g cysteine, and 7 mL ethylenediamine at 70 °C for 1, 3, 5, and 10 h, respectively, as shown in Figure 2e–h. In comparison with the SEM images of Figure 2e,h, the dimensions and lengths of the nanowires were also enlarged with a long aging time. Prolonging the reaction time could lead to an increase in the dimensions and lengths of the Cu_2_S nanowires rather than the growth of a higher density of Cu_2_S nanowires. The results also indicate that the growth of the existing nucleus that has been formed at a certain location is easier than the formation of new nuclei [14,31]. The influence of the volume of ethylenediamine in our systems has also been investigated. Volumes of 4, 7, and 10 mL ethylenediamine were added respectively into the reaction solutions with 10 g sodium hydroxide at 70 °C for 10 h. The SEM images show that a certain amount of ethylenediamine was indispensable for controlling the morphology and density of nanowires, as shown in Figure S1 (see supplementary file). 

**Figure 3:**
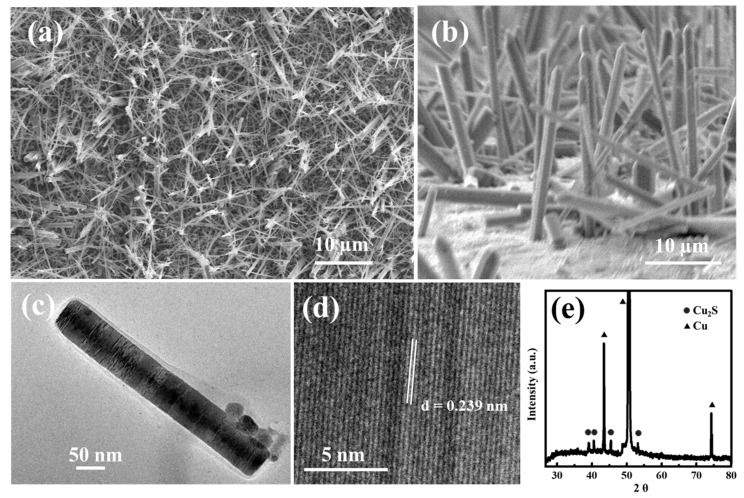
Structural characterizations of Cu_2_S nanowires. The top-view (**a**) and tilt-view (**b**) SEM images of Cu_2_S nanowires. (**c**) High-magnification and (**d**) high-resolution TEM image of Cu_2_S nanowires. (**e**) The XRD patterns of Cu_2_S nanowires.

After comparing these SEM images of Figure 2 and Figure S1 to explore the optimum value of the experiment, high-quality Cu_2_S nanowires were successfully synthesized using the parameters of 10 g NaOH, 1g cysteine, and 7 mL ethylenediamine, which were added respectively into the reaction solution, and finally, it was heated at 70 °C for 10 h. Figure 3a shows a low-magnification SEM image of the as-synthesized high-quality Cu_2_S nanowires grown on the copper substrate. The detailed parameters of this experiment are provided in the Materials and Methods section. The highly ordered Cu_2_S nanowire arrays covered the entire area of the copper substrate compactly and uniformly. Figure 3b is a tilting-angle view SEM image of well-aligned Cu_2_S nanowire arrays, and the average length of the Cu_2_S nanowires is about 30 µm. These nanowires have a quite high length-to-width ratio. The structure and morphology of the nanowires were further studied using transmission electron microscope (TEM), as shown in Figure 3c,d. The Figure 3c indicates a diameter of the Cu_2_S nanowires of close to 50 nm. In Figure 3d, a lattice spacing obtained from a high-resolution TEM image is about 0.239 nm, corresponding to the interplanar distance of the (1 11 1) planes of monoclinic Cu_2_S crystals. The structures of the Cu_2_S nanowire arrays on copper substrate were also determined by means of high-resolution θ-2θ (theta) X-ray diffraction measurements. As shown in Figure 3e, all of the diffraction peaks of the as-grown sample can be ascribed to the single phase of the orthorhombic-structured Cu_2_S crystal phase (JCPDS No. 23-0961) with lattice constants of a = 1.350, b = 2.732, and c = 1.185 nm [14]. It should be noted that there are three additional peaks (2θ = 43.32°, 50.45°, and 74.13°) corresponding to the substrate copper foil. 

### 3.2. ElectronField Emission Property

The materials of interest from field emission have been classified as metallic materials, including carbon nanotubes (CNTs) [32], gold [21], metal silicides [33,34], or wide-bandgap and semiconducting materials such as ZnO [35], WO_3_ [36], and CuO [37] nanowires.

**Figure 4:**
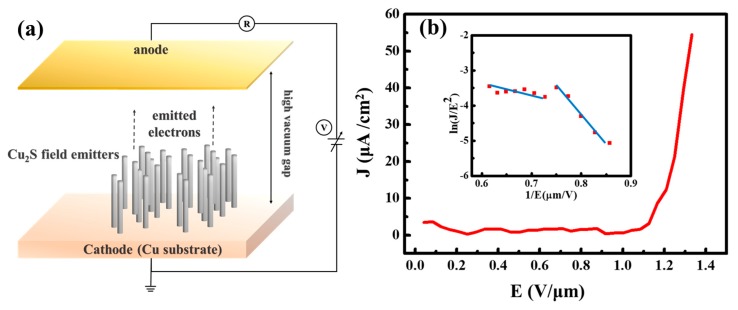
(**a**) Schematic of the field-emission measurement setup. (**b**) Field-emission current (J) versus applied electric field (E) plot measured at 100 μm between cathode and anode separation and pressure of 1 × 10^−7^ torr. Its inset image is the Fowler–Nordheim (F-N) plot of the Cu_2_S nanowires.

The field-emission properties of Cu_2_S nanoemitters were measured, as shown in Figure 4a. The Cu_2_S nanowires vertically grown on Cu substrates show great field-emission properties; the field-emission current density–electrical field (J–E) curve is presented in Figure 4b. The turn-on field is 1.19 V/μm; this value was defined as the applied voltage to produce emitting current density of 10 μA/cm. The field-emission characteristics were theoretically evaluated by the Fowler–Nordheim (F–N) equation (Equation (1)) [38].

(1) J (E)= AE2∅−1exp(−B∅32βE)

where A and B are two constants, corresponding to 1.56 × 10^−10^ [AV^−2^(eV)] and 6.83 × 10^3^ [V (eV^−3/2^) (μm^−1^)], respectively [38]. E is the applied field, β is the field-enhancement factor of the nanowires, ∅ is the work function of the emitter, which is 5.6 eV for Cu_2_S, and J is the current density. The emission behavior can be examined from the linearity of the curve by the corresponding Fowler–Nordheim plot *(ln (J/E^2^)-1/E* curve) of the device after 50 tests (see upper-left inset image in Figure 4b). The maximum emission current can reach up to 55 μA/cm^2^ as the applied voltage can reach over 1000 V. 

The linearity of these curves implies that the field emission from these Cu_2_S nanowires follows the F–N theory and exhibits two negative linear associations in the measured range. At low bias voltages, the field-emission mechanism obeys the properties of the traditional Fowler–Nordheim equation, while the field emission behavior under high bias voltages deviates from the Fowler–Nordheim equation, but a linear relationship on the F–N coordinate still appears. Several reasons have been used to explain the deviation of field emission from Fowler–Nordheim behavior. The deviation from the F–N plot at high bias voltages has frequently been attributed to the space charge effect, localized states, and gas adsorbates [39,40]. 

At the microscopic point, the β values should be experimentally studied. The *β* factor, reflecting the degree of field emission for the tip shape on a planar surface, can be estimated by the slope (−BΦ^3/2^/β) of the F–N plot. At the applied high field, the β value indicates that the barrier-tunneling mechanism is responsible for the field emission. We estimate the β value from the F–N plot. The field enhancement factors β_1_ and β_2_ are 19,381 and 5787, respectively, which are much higher than in the previous report [18,22,32,33,34,35,36,37]. Furthermore, some previous articles have reported that the β value is related to the geometry of the crystal specified by the radius of the curvature of the tip [41,42,43] and other factors, such as the emission height, the crystal structure, conductivity, and the nanostructure density [44].

### 3.3. Photocatalytic Activity

**Figure 5:**
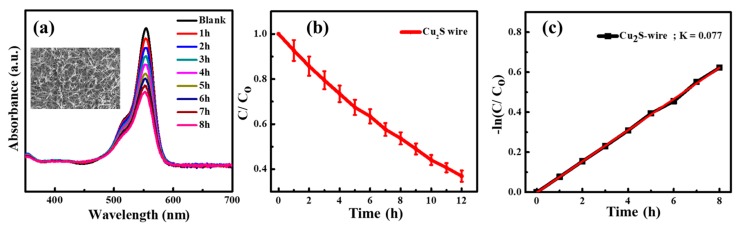
The illustrations display the performance of the photocatalytic activity of the Cu_2_S nanowires over various irradiation periods. (**a**) UV/Vis absorption intensity of the RhB with a series of various times. The upper insert is an SEM image of the experimental sample. (**b**) Photocatalytic degradation of RhB under UV illumination. (**c**) The kinetic formula of RhB photodegradation, K, is a constant of photodegradation rate.

To further measure the photocatalytic properties of the Cu_2_S nanostructured wires, a series of the reactions were investigated for their application of photocatalysis degradation from 1 to 8 h. The results of the photocatalytic activity of the Cu_2_S nanowire arrays are presented in Figure 5. The UV-visible absorbance spectra of the 5 μM rhodamine B (RhB) solution with the Cu_2_S photocatalyst at different irradiation times were measured in Figure 5a. This indicates that Cu_2_S nanowire photocatalysts have an inclination to decrease the UV-visible absorbed wavelength of 554 nm for a longer period of irradiation time. Cu_2_S nanowires have great photocatalytic properties in comparison with the same bulk-type materials due to the larger specific surface area [45,46]. Figure 5b shows that our Cu_2_S nanowires have a degradation rate percentage of 47.4% after continuing testing for 8 h. Figure 5c reveals the photodegradation kinetics of rhodamine B. The photodegradation of dyes follows the first-order reaction described by the Langmuir–Hinshelwood (L-H) mechanism (Equation (2)) [20,47,48].

(2) $$\mathrm{lnC}={\mathrm{lnC}}_{0}-\mathrm{kt}$$

where C_0_ is the initial concentration of the aqueous solution of dyes, C is the corresponding concentration of the aqueous solution of dyes which was measured at various irradiation times, K is a constant of photodegradation rate. After the first-order kinetics calculation, the photodegradation rate constant (K) was obtained by plotting −ln(C/C_0_) as a function of the various irradiation times. The experimental results indicate that surface area would play a significant role in the efficiency owing to the photocatalytic reaction which occurred exactly on the surface. A photocatalyst with a higher specific surface area is important in the enhancement of photocatalytic performance due to more surface active sites and photocatalytic reaction centers.

Generally, the photoelectrochemical performance of semiconductors mainly depends on the generation of the photoinduced electron, separation of electron–hole pairs, and efficiency of charge-carrier transfer. The previous works of literature have shown that enhanced photoactivity of metal chalcogenide semiconductors such as Cu_2_S and CdS [49,50,51] is primarily attributed to the improved lifetime and transfer of photogenerated charge carriers. The value of the specific surface area does not mainly determine the efficiency of the photoactive reaction, and the study of the detailed mechanism still needs further investigations.

## 4. Conclusions

The free-standing, single-crystal Cu_2_S nanowires have been successfully produced on copper metal substrates via a one-step template-free solution route. The morphology of the Cu_2_S nanowires is needlelike with an average length of 30 μm and average diameter of 50 nm. Ethylenediamine plays an important role in electron transfer in redox reactions and in the formation of binary chalcogenide nanowires. The dissolution of chalcogen in ethylenediamine also affects the formation of chalcogenides. In addition, the single-crystalline Cu_2_S nanowires exhibit outstanding properties with a low turn-on field of 1.19 V/μm and high current density of 55 μA/cm^2^ according to the Folwer–Nordheim (F–N) theory, as well as a reproducible value of the field-enhancement factor β_1_ of 19,381 and β_2_ of 5787, respectively. On the other hand, because of their excellent optical performance, the Cu_2_S nanowires are good photocatalysts which also showed effective photocatalytic activity, and RhB can be degraded rapidly with UV illumination at room temperature. The reliability test indicated that the photocatalytic reaction of our Cu_2_S nanowires was highly efficient during periods of continuing specialty testing. Our Cu_2_S nanowires have a degradation rate of 47.4% after an 8 h irradiating reaction. The above outstanding results warrant possible applications for Cu_2_S nanowires as electron field emitters and chemical photocatalysts in future nanoelectronic devices.

## Supplementary Materials

The following are available online at https://www.mdpi.com/2079-4991/10/2/399/s1, Figure S1: SEM images of as-prepared free standing Cu_2_S nanowire grown under different reaction conditions. The three conditions are added (a) 4 mL, (b) 7 mL, (c) 10 mL ethylenediamine with 10 g sodium hydroxide at 70 °C for 10 h.
