# Supplementary material for: Development of Crystalline Cu2S Nanowires via a Direct Synthesis Process and Its Potential Applications
Source: Nanomaterials (Basel). 2020 Feb 24;10(2):399. doi: 10.3390/nano10020399 (PMC7075312; doi:10.3390/nano10020399)
Supplement: Supplementary file 1 [file nanomaterials-10-00399-s001.pdf]

# Supplementary file

# Development of Crystalline Cu2S Nanowires via A Directly Synthesis Process and Its Potential Applications

**Chih-Yen Chen[a,*], Jian-Ru Jiang[b], Wen-Shuo Chuang[a], Ming-Song Liu[b], Sheng-Wei Lee[b,*]**

[a] Department of Materials and Optoelectronic Science, National Sun Yat-Sen University, Kaohsiung 804, Taiwan, ROC

[b] Institute of Materials Science and Engineering, National Central University, Taoyuan 320, Taiwan, ROC

* Correspondence: cychen@mail.nsysu.edu.tw; swlee@g.ncu.edu.tw

The SEM images in Figure S1 reveals the volume of 4 ml, 7ml, and 10 ml ethylenediamine which were added respectively into the reaction solutions with 10 g sodium hydroxide at 70 ℃ for 10 h. Figure S1 show a certain amount of ethylenediamine was indispensable to control the morphology and density of nanowires as shown in Figure S1 (see supplementary file).

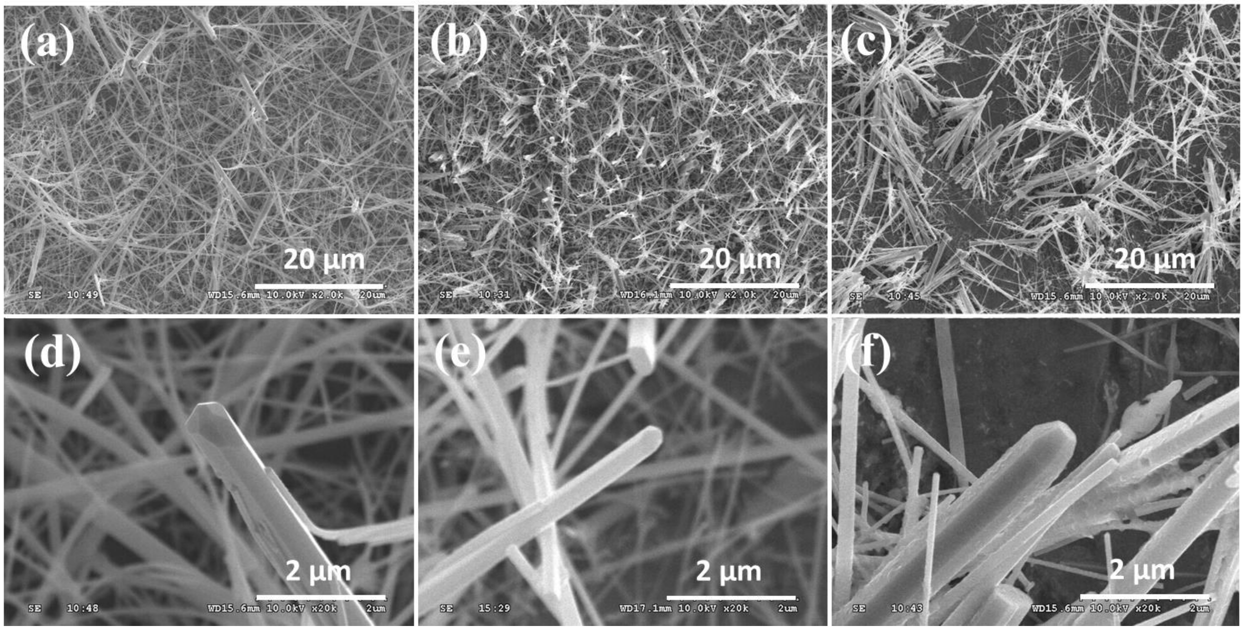


Figure S1. SEM images of as-prepared free standing $Cu_2S$ nanowire grown under different reaction conditions. The three conditions are added (a) 4 ml, (b) 7 ml, (c) 10 ml ethylenediamine with 10 g sodium hydroxide at 70 ℃ for 10 hours.
